# Integrating the Role of UTAUT and TTF Model to Evaluate Social Media Use for Teaching and Learning in Higher Education

**DOI:** 10.3389/fpubh.2022.905968

**Published:** 2022-07-07

**Authors:** Ali Mugahed Al-Rahmi, Alina Shamsuddin, Eta Wahab, Waleed Mugahed Al-Rahmi, Uthman Alturki, Ahmed Aldraiweesh, Sultan Almutairy

**Affiliations:** ^1^Faculty of Technology Management and Business, Universiti Tun Hussein Onn Malaysia, Johor, Malaysia; ^2^Self-Development Skills Department, College of Common First Year, King Saud University, Riyadh, Saudi Arabia; ^3^Educational Technology Department, College of Education, King Saud University, Riyadh, Saudi Arabia

**Keywords:** UTAUT theory, task-technology fit (TTF), social media, academic performance impact, performance expectancy (PE)

## Abstract

Investigation of task-technology fit and intention to use social media tools needs to focus specifically on higher education for teaching and learning, and its impact on students' academic performance. This article aims to develop a model that would identify essential aspects that are predicted to continue to play a large role in TTF for learning in BI, which could be used to improve academic performance in higher education. The purpose of this study was to investigate the characteristics and aspects of SM and the relationship between their use in the TTF and UTAUT theory to determine how they affect research students' satisfaction and AP in HE institutions. Data for the unified theory of acceptance and use of technology (UTAUT) and task-technology fit (TTF) theories were collected using a questionnaire survey. This research hypothesizes that behavioral intention to utilize social media and task-technology fit for learning will influence social characteristics, technology characteristics, performance expectancy, and effort expectancy, all of which will improve academic performance. As a test bed for this research, a structural equation model (SEM) was constructed examining the relationships between factors that affect students' academic performance. A stratified random sample strategy was used to disseminate the main tool of data collection, a questionnaire, to 383 students. A quantitative method was used to examine the results. The obtained outcomes showed that there was a correlation among social characteristics, technological characteristics, behavioral intention to use social media, and task-technology fit for academic performance, which aided student performance and results. The study indicates that PEX and EEX also demonstrated a strong relation to task-technology fit and behavioral intent to use social media for academic purposes, both of which positively impacted academic performance. As a result, the study found that behavioral intention to utilize and task-technology-fit social media promote students' active learning and enable them to discuss and exchange knowledge and information more efficiently. In conclusion, we encourage students to use social media for educational purposes in their studies and teaching through lectures in HE institutions.

## Introduction

The outbreak of corona virus disease 2019 (COVID-19) has caused a global public health emergency. Emergency measures were implemented to prevent the virus from spreading, limiting all non-essential public movements. When educational institutions closed, it became clear that a rapid transition from physical to digital learning was required (1). The COVID-19 pandemic has compelled policymakers, university administrators, and faculty members of institutions of higher education to explore alternatives to the normal classroom-based learning method. Numerous Malaysian colleges, for instance, have pushed faculty members to use free communication tools such as Google Classroom and Zoom. Institutions and faculty members use social media platforms such as Facebook, WhatsApp, and YouTube to interact with their students. Institutions have encouraged faculty members to engage with students using official pages and formal groups on social networking sites (SNSs) such as Facebook and WhatsApp (2). As a result of COVID-19, and for the first time, both instructors and students in many developing countries are required to interact online for academic purposes. In the absence of an online learning management system (LMS), social media can also provide a great opportunity for universities to publicly interact with their students in order to support online learning (3). The study capitalizes on the presence of students and academic staff on social networks to facilitate online social interaction and produce a successful online learning experience (4–7). Academics worldwide have been researching how university and college learners utilize social media (SM), with some findings indicating that it impacts both general learning and instructional efficiency. Its natural fit for enhancing students' oral and written abilities *via* extended practice has also been demonstrated to be useful in learning other languages (8). Student usage of SM in the classroom, on the other hand, has caused academic problems by affecting grade point averages, student satisfaction, and overall academic progress. Students conducting research using SM to learn can find it difficult to focus and manage their time. Analyzing the related literature, researchers found that the time set aside for social engagement on Facebook is not always efficiently employed for educational reasons in a study by **(author?)** (9, 10, 11, 12). Many teachers in Asian countries utilize SM as an informal TTF tool, mostly for social communication rather than to promote student engagement or academic performance (13–15). In a similar vein (3, 16), asserted that there is no relationship between online activities and institutional learning. Student satisfaction and AP were significantly influenced by TTF with the UTAUT theory, which has previously had multiple significant negative effects on student satisfaction and AP (14, 17, 18). Despite varied results, scholars suggest that a thorough understanding of the topic combined with proper implementation of SM will result in more learner-centered educational institutions (19–21).Thus, gaps in this knowledge are intended to be addressed in this research by developing a model for the use of social media in task-technology-fit with behavioral factors that affect students' academic performance in Malaysian higher education. According to research results, a large number of educators in Asian countries utilize social media as a tool for informal cooperation, mostly for communication and social networking purposes, instead of utilizing it for the process of improving students' academic performance (22). Additionally, students use social media infrequently for educational purposes (24). Furthermore, students have a strong desire to collaborate, learn, and communicate using cutting-edge technology; thus, their uniqueness influences can be deceiving to the faith, as social media encourages collaboration and communication (25).Furthermore, a number of academics have examined SM in HE from a variety of perspectives and with diverse goals. As a result, both the TTF and UTAUT aspects that influence students' AP *via* SM should be researched (14, 26). While examining technology acceptance, researchers frequently use UTAUT to investigate how people use technology after accepting reimbursements, as well as factors that influence their decisions. Experts dispute the practical usefulness and theoretical assumptions of the UTAUT theory despite the fact that it is a well-known and frequently cited model (27, 28). In complicated circumstances, social networking sites can leverage the TTF model to build a simple design basis. The TTF model's SM adoption is currently the subject of only a small amount of research. It may not be appropriate for use in SM, because it ignores social structure. The task-technology-fit model was improved by merging the UTAUT theory into this study to overcome this problem. Furthermore, regardless of deliberate considerations, the TTF model focuses on specific implications for task performance. To understand the effects of TTF on user intentions, the proposed framework includes an intent structure. The purpose of this study was to investigate the characteristics and aspects of SM and the relationship between their use in the TTF and UTAUT theory to determine how they affect research students' satisfaction and AP in HE institutions. There is currently no model for assessing student happiness and academic success despite the existence of numerous SM models. As a result, there is a vacuum in research on student satisfaction in higher education when utilizing SM for TTF and UTAUT modeling. As a result, the purpose of this research was to develop a model that would identify essential aspects that are predicted to continue to play a large role in TTF for learning in BI, which could be used to improve academic success in higher education.

## Research Model and Hypothesis Development

**Figure 1:**
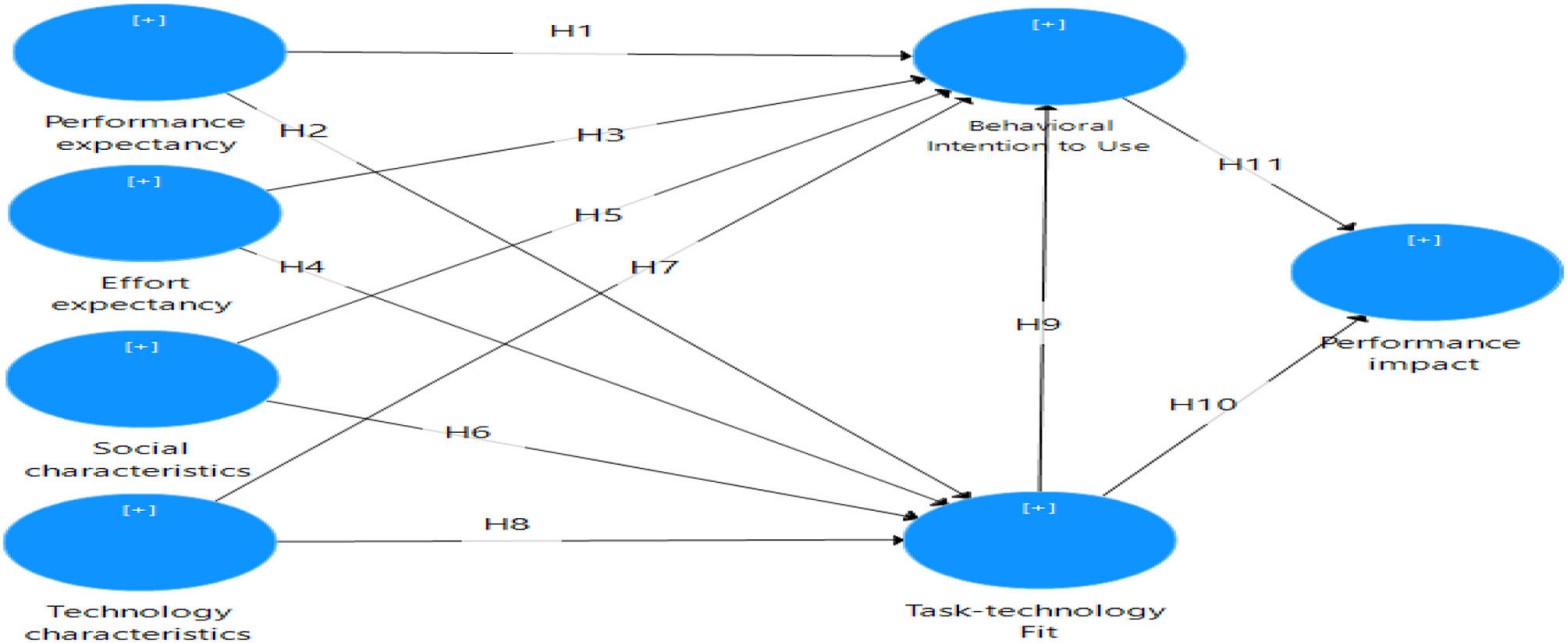
Research model and hypotheses (source: authors).

The theoretical model suggested in this study investigates all TTF theory-related aspects such as social characteristics (SCs) and technological characteristics (TCs), and UTAUT theory-related factors such as PEX, EEX, and FC. This section discusses aspects that have been discovered to influence learner satisfaction (SS) and academic performance (AP) in educational institutions (see Figure 1).

### Performance Expectancy

Performance expectancy (PEX) is defined as “the degree to which an individual believes that using the system will assist him or her in obtaining improvements in work performance” (29). According to UTAUT, PEx is one of the direct predictors of behavioral intention (BI) to utilize technology. Following the facilitating conditions, detailed information regarding BI is provided. PE has been proven to be a significant factor in BI in various studies. According to (30), they conducted research with university students from Qatar and the United States on teaching and learning adoption. In both samples, PE was revealed to be one of the important predictors of BI. Similarly (31), used UTAUT to study factors impacting university students' and educators' acceptance of SM platforms.

H1: PEX will have a substantial effect on TTF.
H2: PEX will have a substantial effect on BI.

### Effort Expectancy

The effort expectancy (EEX) construct measures how easy a system is to use (29). It expresses instructors' conviction that using social media in the classroom would benefit both them and their pupils (32). According to UTAUT, EE is one of the direct determinants of BI. EE has been found to be a significant factor in BI in numerous investigations. For example (33), used UTAUT to investigate pre-service teachers' self-reported intentions to use technology. They discovered that EE was a key factor in BI's decision to embrace information technology. Within the margins of UTAUT (34), conducted a study to explore undergraduate students' readiness for online learning and extend the model with numerous variables.

H3: EEX will have a substantial effect on TTF.
H4: EEX will have a substantial effect on BI.

### Social Characteristics

In earlier SM studies, social characteristics (SOCs) were used as mediators to study the causality between input and output parameters. According to (35), social influence was used as a facilitator to look into user donations in diverse virtual cultures. Social capital has been used as a mediator in a number of studies to show user intentions and behaviors. For instance (36), studied the impact of “trust” on influencing a user's readiness to consent to something or someone. Moreover (37, 38), on the other hand, investigated the function of social ties and social evasion in determining team cohesion. Hsiao et al. (39) investigated user contributions in virtual societies using social effect as a mediator. The researchers propose the following hypotheses in light of the foregoing debate:

H5: Social characteristics will have a substantial effect on TTF.
H6: Social characteristics will have a substantial effect on BI.

### Technology Characteristics

The term “technology characteristics” (TCs) describes how technology can be used to achieve the best fit (40). Facebook, LinkedIn, and Twitter, i.e., were designed with specific goals and purposes in mind for specific user groups. These methods can be used to demonstrate the impact of causal priming on predicted behavior (41). We investigated how active usage of Twitter facilitates communication with others and discovered that various Twitter features can assist with this. Five mediating effects of social media, such as use of tools (Facebook, Twitter, WeChat, etc.) as a social networking platform, were also looked into. When task characteristics and task performance were evaluated, media intake was found to improve task performance (42). Al-Rahmi et al. (43) investigated the impact of tool integration on information and system quality predictions. Wang and Lin (44) identified TTF and the distribution of team performance on repeated activities, whereas The TTF model was used to study factors that influence individual performance in enterprise resource planning (45). They merged two different types of technological characteristics into their frame. In light of the above debate, the researcher proposes the following hypotheses:

H7: TCs will have a significant effect on TTF.
H8: TCs will have a significant effect on BI.

### Task-Technology Fit

The TTF model is based on the relationship between TCs and task specifications (46). According to the model, focusing merely on learners' expectancy of knowledge is insufficient for estimating its uptake. Learners will accept technology if they consider it will help them do their daily tasks effectively (7, 47). The paradigm of this theory throws light on the practical elements of technology use. The TTF model forecasts that users will adopt a technology based on the relationship between technological features and performance expectancy (7, 47), because focusing on student anticipations of technology is inadequate. The researchers propose the following hypotheses in light of the preceding discussion:

H9: TTF will have a substantial effect on BI.
H10: TTF will have a substantial effect on PI.

### Behavioral Intention to Use Social Media

Behavioral intention (BI) to use is defined as a personal desire to continue or use technology, including the variables that influence technology use, and the factor that determines the usage of a technology (48). As a result, learners' BI to use SM to learn and online communication would enrich their learning performance in this study. Furthermore, our research identifies the use of SM for increased cooperative learning as a key component in developing technology-based theories (49, 50). All of these theories arose from the foundation of TRA, which believes that SM use is a function of one's attitude toward particular norms; subsequently, this was extended to involve apparent control, referred to as TPB (50). Furthermore, PU and PEOU are thought to be post-adoption beliefs of a regular user, leading to increased user delight and continuing intent (51). Some researchers have discovered that people who enjoy using SM are more likely to consider their involvement with the system as a whole and create better SM usage habits (52, 53). According to (19, 50), BI is related to students' intention to both use SM regularly and embrace the application in the future, or not to use it. BI refers to how students would use SM applications for collaborative learning in the future in this study. Al-Rahmi et al. (54) found that BI directly impacts the use of SM for collaborative learning. Aside from that, the key reason for developing technology-based models and theories is the desire of users to use SM on any system (23, 55).

H11: BI will have a substantial effect on PI.

### Academic Performance

Academic performance (AP) can be characterized as a learning outcome in which a student, a learner, an instructor, or an institution has achieved their educational goals (56). According to Junco and Cotten (57), SM impacts students' educational achievements in various domains of research. It has been noted that forming a Facebook-oriented social group might help students develop more smoothly (58). However, there are a few exceptions where the data reveal a favorable association between Twitter and Facebook (59), as well as integration to broaden learning (60). According to (61), SM serves as a platform for engagement, communication, and collaboration among research students and professors in their departments. Furthermore, according to (62), SM has little or no impact on students' AP. Furthermore, Wang et al. (63) attempted to investigate the relationship between students' AP and their use of Facebook. Their findings demonstrated a significant unfavorable relationship between students' use of Facebook and their AP. In comparison to non-users, students revealed that they spend less time per week studying regularly. The majority of students required that they utilize their Facebook accounts at least once a day. This agrees with (9, 57). According to studies examining the effects of SM use on students' AP, all students believe that it is suitable for their mentors to have a Facebook presence, where both professors and students get socialized (64). Furthermore, the use of SM networks aids in the development of a favorable relationship between students' AP and satisfaction (65).

## Methods and Materials

### Design of the Study

This survey study aims to report findings on students' use of SM for learning in educational institutions. The study included one primary endogenous construct, namely, the AP impact. The proposed model shown in Figure 1 consists of seven constructs: PE, EE, BI to use, SOC, TC, TTF, and PI. Eleven path lines were proposed for the seven constructs; two-path lines were proposed for PE, EE, SOC, TC, and TTF. One path line was proposed for BI to use, all were hypothesized to significantly predict seven constructs (Figure 1).

### Measurement Instruments

An instrument was adopted from previous research. There are seven constructs with thirty-five indicators, as shown in Table 1. Performance expectancy (PEX) was proposed with the establishment of five items recommended by (66). Effort expectancy (EEE) was proposed with the establishment of five items recommended by (66). Social characteristics were proposed with the establishment of five items recommended by (67, 68). Technology characteristics (TCs) were proposed with the establishment of five items recommended by (67, 68). Task-technology-fit (TTF) was proposed with the establishment of five items recommended by (67, 68). Behavioral intention (BI) to use was proposed to establish five items recommended by (66, 69). Academic performance impact (PI) was proposed with the establishment of five items recommended by (70). Finally, the instrument's distribution was conducted with thirty-five indicators remaining for the main data collection.

**Table 1:** Constructs and items.

**Construct**	**Items**
Performance expectancy (PEX)	PEX 1—PEX 5
Effort expectancy (EE)	EE 1—EE 5
Social characteristics (SC)	SC 1—SC 5
Technology characteristics (TEC)	TEC 1—TEC 5
Task-technology fit (TTF)	TTF 1—TTF 5
Behavioral intention to use (BI)	BI 1—BI 5
Academic performance impact (PI)	PI 1—PI 5

### Measurement Instrument Data Collection and Analysis

**Figure 2:**
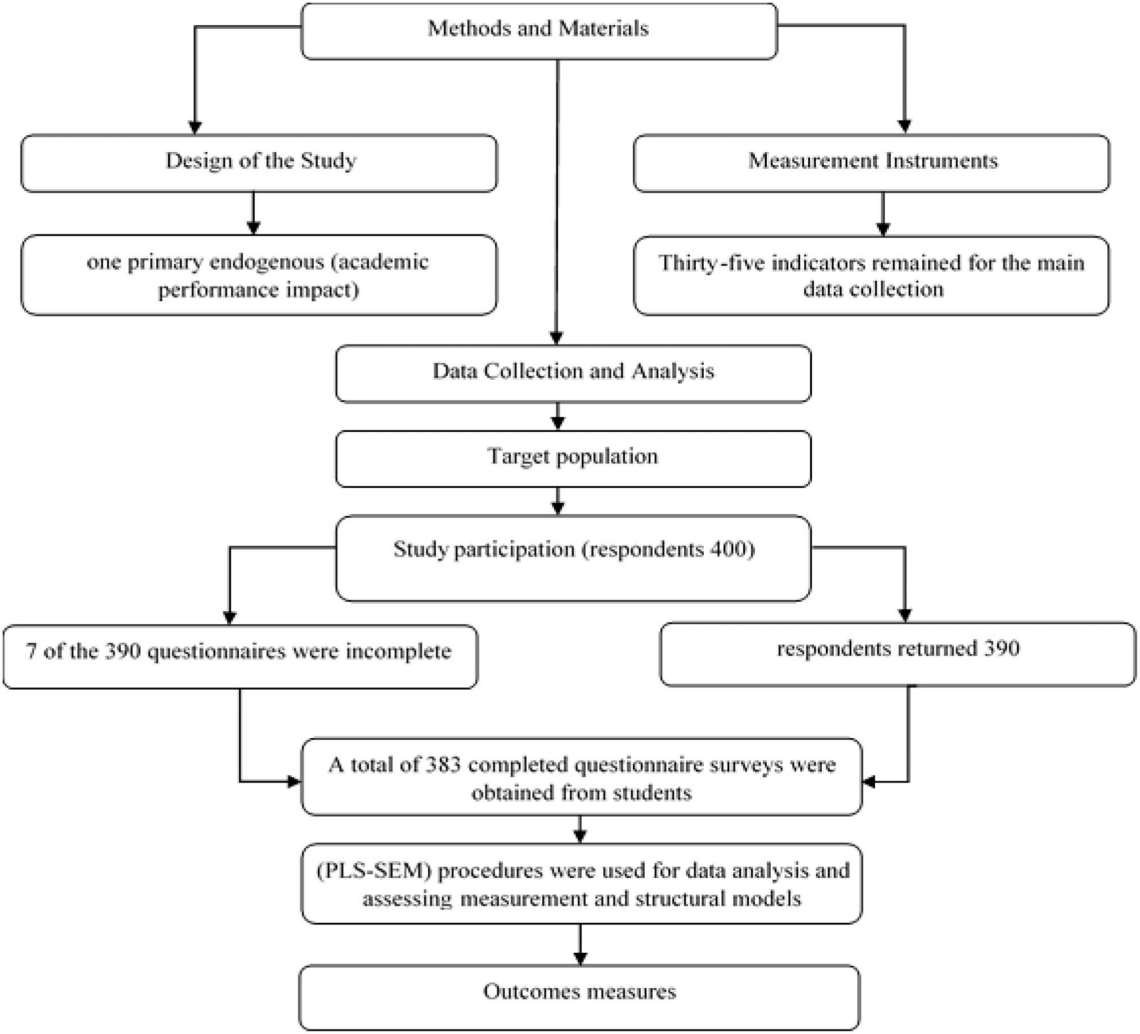
Diagram for methods and materials.

We distributed 400 questionnaires for the study, of which respondents returned 390; after a manual analysis, 7 of the 390 questionnaires were incomplete (“students did not finish the survey”) and had to be dropped, leaving 383. Such exclusions were recommended by Hair et al. (71), who stated that outliers could lead to inaccuracies. To evaluate the impact of adopting the model for teaching and learning in educational institutions, we built a conceptual model combining the UTAUT and TTF theories. The purpose of this study was to find out what students thought about using SM for TTF and BI to quantify the influence of AP in HE. The questionnaire was handed out by hand at UTHM. Respondents were asked to fill it out anonymously to provide feedback on TTF and BI's use of SM and their perceptions of SM's impact on AP and educational sustainability. IBM SPSS Statistics version 26 and Smart-PLS 3.3.3 were used to analyze the data, as well as structural equation modeling and the diagram for Methods and materials (Figure 2).

A total of 383 completed questionnaire surveys were obtained from students, including 305 (79.6%) from men and 78 (20.4%) from women; 123 (32.1%) of those who responded were between the age of 18 and 22, 144 (37.6%) were between the age of 23 and 29, 93 (24.3%) were between the age of 30 and 35, 19 (5%) were between the age of 36 and 40, and 4 (1%) were over 41 (refer to Table 2). Partial least square structural equation modeling (PLSSEM) procedures were used for data analysis. In this study, the Smart-PLS 3.3.3 software was utilized for assessing measurement and structural models. Data validity and reliability were measured during their computation in the measurement model. To examine the validity of the data, we reported convergent and discriminant validity. Convergent validity was reported through AVE, whose value should be 0.5; discriminant validity was addressed based on computation processes of Fornell–Larcker criterion, cross-loading, and (HTMT). Meanwhile, to report the reliability of the data, an internal consistency and reliability process was carried out. There were two approaches to reliability; both values should be >0.7. For the assessment model, we reported the significance of the relationship through path coefficient, *t*-value, and *p*-value.

**Table 2:** Constructs, items, and references.

**Items**	**Description**	***N***	**%**	**Cumulative %**
Gender	Male	305	79.6	79.6
	Female	78	20.4	100.0
Age	18–22	123	32.1	37.1
	23–29	144	37.6	74.7
	30–35	93	24.3	99.0
	36–40	19	5.0	5.0
	41–Above	4	1.0	100.0
Specialization	Social science	58	15.1	15.1
	Engineering	124	32.4	47.5
	Science & technology	83	21.7	69.2
	Management	98	25.6	94.8
	Others	20	5.2	100.0
Use _SM	Several times a day	215	56.1	57.7
	An once in a day	100	26.1	100.0
	Several times in a month	62	16.2	73.9
	An once in a month	6	1.6	1.6

## Results and Analysis

### Measurement Model

Hair et al. (72) encouraged four assessments of measurement models for PLSSEM that included consistency reliability, discriminant validity, convergent validity, and indicator loadings.

### Reflective Indicator Loadings

Reflective indicator loadings achieved in the SEM should be >0.7 according to Hair et al. (72). From the computation, all the loadings were higher than 0.7. The highest loading referred to by PE was PEX3 (0.891), while the lowest loading was achieved by SOC (0.738). Thirty-five indicators were included for the next data analysis process (Table 3).

**Table 3:** Constructs, items, IL, CR, CA, and AVE.

**Construct**	**Code**	**Loading**	**CA**	**CR**	**AVE**
Performance	PEX1	0.750	0.890	0.920	0.696
expectancy	PEX2	0.863			
(PEX)	PEX3	0.891			
	PEX4	0.861			
	PEX5	0.800			
Effort	EEX1	0.847	0.906	0.930	0.726
expectancy	EEX2	0.855			
(EE)	EEX3	0.874			
	EEX4	0.868			
	EEX5	0.816			
Social	SC 1	0.858	0.887	0.917	0.690
characteristics	SC 2	0.850			
(SC)	SC 3	0.854			
	SC 4	0.848			
	SC 5	0.738			
Technology	TEC 1	0.869	0.912	0.935	0.742
characteristics	TEC 2	0.874			
(TEC)	TEC 3	0.889			
	TEC 4	0.875			
	TEC 5	0.795			
Task-	TTF 1	0.779	0.867	0.904	0.653
technology	TTF 2	0.845			
fit (TTF)	TTF 3	0.795			
	TTF 4	0.839			
	TTF 5	0.782			
Behavioral	BI 1	0.785	0.875	0.909	0.667
intention	BI 2	0.808			
to use (BI)	BI 3	0.815			
	BI 4	0.843			
	BI 5	0.831			
Academic	PI 1	0.798	0.847	0.891	0.621
performance	PI 2	0.834			
impact (PI)	PI 3	0.777			
	PI 4	0.766			
	PI 5	0.763			

### Internal Consistency Reliability

ICR is implemented to evaluate the consistency of results across indicators. In the current approach, CA and CR were reported. The ICR values should be from 0 to 1. CA and CR ought to be >0.7 according to Hair et al. (72). Table 3 presents the reports of CA and CR. The composite reliability and the CA values for all constructs are sufficient, exceeding the recommended amount. EE had a CA of 0.89 and a CR of 0.92, SOC had a CA of 0.887 and a CR of 0.917, TC had a CA of 0.912 and a CR of 0.935, TTF had a CA of 0.867 and a CR of 0.904, BI to use had a CA of 0.875 and a CR of 0,909, and AP impact had a CA of 0.847 and a CR of 0.891 (refer to Table 3).

### Convergent Validity

Convergent validity is related to construct validity, which means that tests with same or similar constructs should be highly related (72). The convergent validity in this study is reported by calculation of AVE. We applied Smart-PLS 3.3.3 to calculate the AVE (72). Through the algorithm, AVE values ought to be 0.5 or greater (Table 3). From the computation, all the constructs obtained AVE values explaining more than 0.5 of the variances. The PE AVE value was 0.696, EE AVE was 0.726, SOC AVE was 0.69, TC AVE was 0.742, TTF AVE was 0.653, the BI AVE value was 0.667, and PI was 0.621.

### Discriminant Validity

Discriminant validity refers to the degree to which a construct is empirically distinct from other constructs. Three approaches were used in this study to examine the discriminant validity, namely cross loadings (refer to Table 5), HTMT (refer to Table 6), and the Larcker criterion (refer to Table 4). For the Fornell–Larcker criterion, a construct's AVE should be greater than others' shared variance (73). Table 4 shows that the values of the constructs are greater than each construct's shared variances. For instance, the value of PI (0.788) is higher than all of its shared variances: PE (0.51); effort expectancy (0.435), and BI to use (0.588). Discriminant validity was established based on the Fornell–Larcker criterion. Besides, discriminant validity emerges if an indicator loading on a cross loading is smaller than its construct (72).

**Table 4:** Fornell-larcker criterion.

	**BI**	**EEX**	**PEX**	**PI**	**SC**	**TTF**	**TEC**
Behavioral intention to use	0.817						
Effort expectancy	0.570	0.852					
Performance expectancy	0.734	0.542	0.835				
Performance impact	0.588	0.435	0.510	0.788			
Social characteristics	0.499	0.338	0.391	0.494	0.831		
Task-technology fit	0.555	0.543	0.486	0.523	0.495	0.808	
Technology characteristics	0.474	0.385	0.421	0.513	0.410	0.479	0.861

**Table 5:** Measures for cross-loading and loading.

**Factors**	**Code**	**BI**	**EEX**	**PEX**	**PI**	**SC**	**TEC**	**TTF**
Behavioral intention to use	BI_1	**0.785**	0.573	0.631	0.423	0.372	0.326	0.443
	BI_2	**0.808**	0.409	0.574	0.512	0.410	0.400	0.436
	BI_3	**0.815**	0.425	0.538	0.497	0.399	0.342	0.479
	BI_4	**0.843**	0.468	0.619	0.482	0.408	0.403	0.435
	BI_5	**0.831**	0.454	0.631	0.487	0.446	0.460	0.475
Effort expectancy	EEX_1	0.453	**0.847**	0.511	0.350	0.239	0.308	0.432
	EEX_2	0.466	**0.855**	0.461	0.367	0.204	0.280	0.404
	EEX_3	0.499	**0.874**	0.438	0.399	0.289	0.311	0.477
	EEX_4	0.514	**0.868**	0.445	0.355	0.341	0.335	0.479
	EEX_5	0.491	**0.816**	0.457	0.381	0.353	0.398	0.509
Performance expectancy	PEX_1	0.652	0.429	**0.750**	0.490	0.374	0.344	0.415
	PEX_2	0.623	0.461	**0.863**	0.424	0.340	0.380	0.405
	PEX_3	0.617	0.463	**0.891**	0.428	0.321	0.347	0.430
	PEX_4	0.616	0.458	**0.861**	0.432	0.309	0.406	0.383
	PEX_5	0.541	0.445	**0.800**	0.341	0.278	0.268	0.390
Performance impact	PI_1	0.457	0.331	0.376	**0.798**	0.415	0.432	0.406
	PI_2	0.460	0.347	0.396	**0.834**	0.412	0.436	0.422
	PI_3	0.395	0.302	0.363	**0.777**	0.369	0.397	0.415
	PI_4	0.464	0.372	0.411	**0.766**	0.391	0.374	0.417
	PI_5	0.528	0.357	0.456	**0.763**	0.359	0.383	0.399
Social characteristics	SC_1	0.423	0.269	0.293	0.452	**0.858**	0.360	0.440
	SC_2	0.391	0.266	0.317	0.397	**0.850**	0.324	0.401
	SC_3	0.374	0.258	0.311	0.420	**0.854**	0.342	0.394
	SC_4	0.430	0.282	0.337	0.398	**0.848**	0.298	0.414
	SC_5	0.445	0.326	0.363	0.381	**0.738**	0.373	0.399
Technology characteristics	TEC_1	0.418	0.303	0.346	0.409	0.368	**0.869**	0.412
	TEC_2	0.415	0.333	0.378	0.440	0.403	**0.874**	0.412
	TEC_3	0.393	0.300	0.331	0.427	0.326	**0.889**	0.395
	TEC_4	0.364	0.335	0.356	0.448	0.337	**0.875**	0.434
	TEC_5	0.445	0.382	0.395	0.481	0.326	**0.795**	0.407
Task-technology fit	TTF_1	0.462	0.560	0.473	0.379	0.342	0.410	**0.779**
	TTF_2	0.453	0.459	0.398	0.438	0.404	0.403	**0.845**
	TTF_3	0.414	0.383	0.377	0.391	0.348	0.358	**0.795**
	TTF_4	0.470	0.395	0.389	0.445	0.405	0.383	**0.839**
	TTF_5	0.440	0.389	0.325	0.457	0.495	0.379	**0.782**

*The bold values indicate the values which are acceptable*.

**Table 6:** Heterotrait–monotrait (HTMT, <0.9) ratio for discriminant validity.

	**BI**	**EEX**	**PEX**	**PI**	**SC**	**TTF**	**TEC**
Behavioral intention to use							
Effort expectancy	0.639						
Performance expectancy	0.829	0.604					
Performance impact	0.68	0.495	0.582				
Social characteristics	0.564	0.372	0.438	0.57			
Task-technology fit	0.637	0.608	0.552	0.609	0.562		
Technology characteristics	0.528	0.42	0.464	0.583	0.454	0.537	

### Structural Model

#### Collinearity

The assessment of the structural model involved the examination of the model's predictive capabilities. However, before reporting the structural model, the collinearity value should be noted by reporting the variance inflation factor (VIF) values. Notably, the sets of predictors were assessed for collinearity (72), TTF as a predictor of BI to use and PI, EE, PE, SOC, and TC are predictors of BI to use and TTF (Table 7). The values of VIF should not be >3; values exceeding 3 are often considered to have multicollinearity problems. As shown in Table 7, all VIFs are lower than 3.

**Table 7:** Variance inflation factor (VIF).

	**BI**	**EEX**	**PEX**	**PI**	**SC**	**TTF**	**TEC**
Behavioral intention to use				1.445			
Effort expectancy	1.667					1.496	
Performance expectancy	1.622					1.588	
Performance impact							
Social characteristics	1.429					1.308	
Task-technology fit	1.833			1.445			
Technology characteristics	1.441					1.368	

### Analysis of the Structural Model

**Figure 3:**
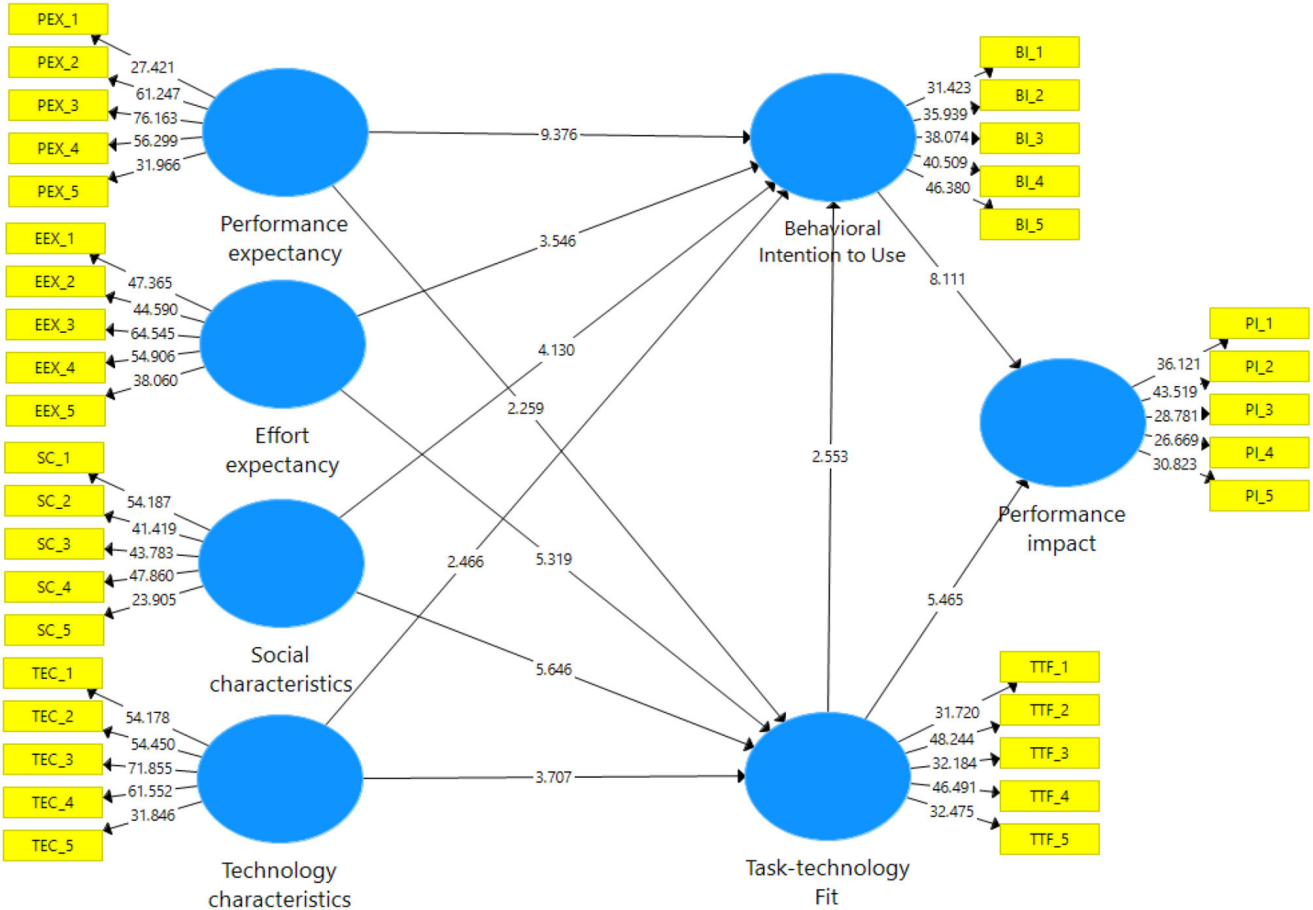
Path *t*-value findings.

For the structural model, the significance of all direct effects or hypotheses was assessed by examining the path coefficients, *t*-statistics, and *p*-values. The results of the bootstrapping computation are presented in Table 8 and Figure 3. Table 8 summarizes the study's findings, including all partnerships. For the relationship PE-> BI to use (H1) (β = 0.498; *t* = 9.376, *p* < 0.001), the hypothesis was accepted. For the relationship PE-> TTF (H2) (β = 0.137; *t* = 2.259, *p* < 0.001), the hypothesis was accepted. H3 and H4 were reported to be significant in influencing EE-> BI to use (β = 0.153, *t* = 3.546, *p* < 0.001) and EE-> TTF (β = 0.305, *t* = 5.319; *p* < 0.001), and the hypotheses were accepted. Moreover, H5 and H6 were reported to be significant in influencing between SOC-> BI to use (β = 0.163, *t* = 4.13, *p* < 0.001) and SOC-> TTF (β = 0.256; *t* = 5.646, *p* < 0.001), and the hypotheses were supported. For the relationship TC-> BI to use (H7) (β = 0.087, *t* = 2.466, *p* < 0.001), the hypothesis was accepted. TC was also a significant predictor for TTF (H8) (β = 0.199, *t* = 3.707, *p* < 0.001), and the hypothesis was accepted. H9 and H10 were reported to be significant in influencing TTF-> BI to use (β = 0.107, *t* = 2.553, *p* < 0.001) and TTF-> PI (β = 0.284, *t* = 5.465, *p* < 0.001), and the hypotheses were accepted. Finally, BI to use was also a significant predictor for PI (H11) (β = 0.431, *t* = 8.111, *p* < 0.001), and the hypothesis was accepted.

**Table 8:** Hypothesis testing.

**Path of hypotheses**	**Path (β)**	***T-*Value**	***P*-values**	**Results**
Performance expectancy -> behavioral intention to use (H1)	0.498	9.376	0.000	Accepted
Performance expectancy -> task-technology fit (H2)	0.137	2.259	0.000	Accepted
Effort expectancy -> behavioral intention to use (H3)	0.153	3.546	0.000	Accepted
Effort expectancy -> task-technology fit (H4)	0.305	5.319	0.000	Accepted
Social characteristics -> behavioral intention to use (H5)	0.163	4.13	0.024	Accepted
Social characteristics -> task-technology fit (H6)	0.256	5.646	0.000	Accepted
Technology characteristics -> behavioral intention to use (H7)	0.087	2.466	0.000	Accepted
Technology characteristics -> task-technology fit (H8)	0.199	3.707	0.011	Accepted
Task-technology fit -> behavioral intention to use (H9)	0.107	2.553	0.000	Accepted
Task-technology fit -> performance impact (H10)	0.284	5.465	0.014	Accepted
Behavioral intention to use -> performance impact (H11)	0.431	8.111	0.000	Accepted

## Discussion

In theory, this research increased the understanding of how to utilize SM platforms for educational purposes by constructing a research model that focused on the importance of BI to use and TTF, PE, EE, social characteristics, technology characteristics, TTF, BI to use, and AP impact as determinants in the study model. As a result, considering BI for education strategy, the study model finds UTAUT and TTF have the most significant impact on AP. As a result, the study's outcomes substantially support the PE variable, confirming H1 and H2, demonstrating that PE has a beneficial influence on BI to use and TTF. To put it another way, when BI to use and TTF are both advantageous and acceptable, PE increases their use. A lot of academics have looked at the significance of PE in the realm of SM use. This result is as expected, because when students perceive that a social media tool is useful to them, their intention to use the tool increases. As a result, the outcomes of this study confirm prior connections between factors, confirming that UTAUT constructs such as PE and EE sway students' outlooks toward technology-enabled learning in higher education (74–77).

Furthermore, the study's findings strongly support the EE component, confirming H3 and H4, implying that EE has a considerable influence on BI to use and TTF in educational institutions. On the other hand, this result can be explained by the strong use of social media tools among students in Malaysian universities, where there are enough students for them to have a significant impact on their peers. As a result, the findings of this study corroborate previous connections between factors (74, 75, 77). Similarly, the study's outcomes substantially support the social and technology characteristics components, confirming H5, H6, H7, and H8, suggesting that social and TC positively impact BI to use and TTF. To put it another way, when BI to use and TTF are both acceptable, the social and technological aspects contribute to enhanced BI to use and TTF for embracing SM in educational institutions. Therefore, TTF and BI to use social media are related to perceptions of social characteristics and technological characteristics, all of which improve student academic activities by obtaining important resources from their peers, including their instructors' guidelines. Experimental evidence suggests that on-campus students need additional support beyond short face-to-face talks when using social media to collaborate. Several academics have looked at the importance of social and TC in the field of using SM. According to the outcomes of this study, using SM improves social and TC, as well as TTF, all of which can increase learners' AP impact, as reported in past and current studies (78–80). BI to use SM is related to perceptions of PE, EE, social characteristics, and TCs, which help students achieve academic performance by providing them with important resources from their classmates and their teachers' recommendations. When using SM to communicate, experimental research reveals that on-campus learners require more help beyond short-face-to-face conversations. Furthermore, it has been discovered that for educational purposes, BI to use SM is significantly more useful than “face-to-face” sessions (6, 59, 81) based on advancements connected to instructors' research skill improvement and conception of student exchanges. This investigation has several consequences based on the model and results. The first implication concerns the value of agreed-upon structures. In the relationship among PE, EE, social factors, and function of TTF in using SM as a source of learning, TCs are especially significant. Second, a faculty can demonstrate how to utilize technology by giving students educational tools that might assist them in learning how to do so, bearing in mind that SM should be viewed as both performance and an EE. Third, learners ought to be informed about the numerous benefits of using SM platforms and given course content or other learning objectives connected to long-term learning that will improve their academic performance.

## Conclusion

According to the findings of this study, two types of TCs will have an impact on AP *via* TTF for learning. The purpose of this research was to develop a model that would identify essential aspects that are predicted to continue to play a large role in TTF for learning in BI, which could be used to improve academic success in higher education. According to the results, increasing BI to use SM for learning objectives and the PE and EE of SM impacted AP. The data also showed that students' BI to use SM positively impacted TTF and educational performance. Furthermore, according to the findings, TTF suited for using SM positively impacted BI to use and educational outcomes. Similarly, the outcomes also indicated that academic performance impact is affected by increasing the TTF and BI to use social media for learning purposes, as well as the performance expectancy, effort expectancy, social characteristics, and technological characteristics of social media. The TTF theory was also used to assess students' intentions to utilize SM for learning to improve their AP in HE, proving the UTAUT hypothesis. To summarize, TTF and BI to use SM can help students with their learning activities, knowledge sharing, information exchange, and peer dialogues. Even though this study followed a thorough research process, some potential flaws could be detected and investigated in future research. The quantitative outcome may not reflect each respondent's full understanding of the research question. A qualitative approach would be extremely beneficial in strengthening the study's conclusions. Because all the respondents in our sample came from the same university, future studies would need to include more people taking various majors. Because the sample lacked qualitative data, it was forced to rely on students' expectations, which may differ from professors'. Future studies are suggested to increase data collection from universities or school students in other states or repeat the research in other provinces rather than Malaysia because of environment's dissimilarity.

## Data Availability Statement

The original contributions presented in the study are included in the article/supplementary material, further inquiries can be directed to the corresponding author/s.

## Ethics Statement

Ethics review and approval/written informed consent was not required as per local legislation and institutional requirements.

## Author Contributions

AA-R, AS, and EW: conceptualization, methodology, resources, data curation, and project administration. AA-R, WA-R, SA, and AA: software. AA-R, AS, EW, UA, and AA: validation. AA-R, AS, EW, and WA-R: formal analysis. AA-R: investigation. AA-R, WA-R, SA, and AS: writing-original draft preparation and writing-review and editing. AA-R, AS, EW, WA-R, UA, SA, and AA: visualization. AS, WA-R, and EW: supervision. All the authors have read and agreed to the published version of the manuscript.

## Funding

The authors extend their appreciation to the Deanship of Scientific Research at King Saud University for funding this work through research group (No. RGP-1435-033).

## Conflict of Interest

The authors declare that the research was conducted in the absence of any commercial or financial relationships that could be construed as a potential conflict of interest.

## Publisher's Note

All claims expressed in this article are solely those of the authors and do not necessarily represent those of their affiliated organizations, or those of the publisher, the editors and the reviewers. Any product that may be evaluated in this article, or claim that may be made by its manufacturer, is not guaranteed or endorsed by the publisher.
